# Supplementary material for: Longitudinal Speckle Tracking Strain Abnormalities in Chagas Disease: A Systematic Review and Meta-Analysis
Source: J Clin Med. 2022 Jan 31;11(3):769. doi: 10.3390/jcm11030769 (PMC8846382; doi:10.3390/jcm11030769)
Supplement: Supplementary file 1 [file jcm-11-00769-s001.zip › jcm-1507241-supplementary.pdf]

Supplementary file

Table S1. PRISMA Checklist.

Section/topic	#	Checklist item	Reported on page #
**TITLE**			
Title	1	Identify the report as a systematic review, meta-analysis, or both.	1
**ABSTRACT**			
Structured summary	2	Provide a structured summary including, as applicable: background; objectives; data sources; study eligibility criteria, participants, and interventions; study appraisal and synthesis methods; results; limitations; conclusions and implications of key findings; systematic review registration number.	2
**INTRODUCTION**			
Rationale	3	Describe the rationale for the review in the context of what is already known.	3
Objectives	4	Provide an explicit statement of questions being addressed concerning participants, interventions, comparisons, outcomes, and study design (PICOS).	4
**METHODS**			
Protocol and registration	5	Indicate if a review protocol exists, if and where it can be accessed (e.g., Web address), and, if available, provide registration information including registration number.	4
Eligibility criteria	6	Specify study characteristics (e.g., PICOS, length of follow-up) and report characteristics (e.g., years considered, language, publication status) used as criteria for eligibility, giving rationale.	4
Information sources	7	Describe all information sources (e.g., databases with dates of coverage, contact with study authors to identify additional studies) in the search and date last searched.	Supplement
Search	8	Present full electronic search strategy for at least one database, including any limits used, such that it could be repeated.	Supplement
Study selection	9	State the process for selecting studies (i.e., screening, eligibility, included in systematic review, and, if applicable, included in the meta-analysis).	4
Data collection process	10	Describe method of data extraction from reports (e.g., piloted forms, independently, in duplicate) and any processes for obtaining and confirming data from investigators.	4
Data items	11	List and define all variables for which data were sought (e.g., PICOS, funding sources) and any assumptions and simplifications made.	4
Risk of bias in individual studies	12	Describe methods used for assessing risk of bias of individual studies (including specification of whether this was done at the study or outcome level), and how this information is to be used in any data synthesis.	4, 5
Summary measures	13	State the principal summary measures (e.g., risk ratio, difference in means).	5

Synthesis of results	14	Describe the methods of handling data and combining results of studies, if done, including measures of consistency (e.g., I^2) for each meta-analysis.	5
Risk of bias across studies	15	Specify any assessment of risk of bias that may affect the cumulative evidence (e.g., publication bias, selective reporting within studies).	4, 5
Additional analyses	16	Describe methods of additional analyses (e.g., sensitivity or subgroup analyses, meta-regression), if done, indicating which were pre-specified.	5
**RESULTS**			
Study selection	17	Give numbers of studies screened, assessed for eligibility, and included in the review, with reasons for exclusions at each stage, ideally with a flow diagram.	5, 6
Study characteristics	18	For each study, present characteristics for which data were extracted (e.g., study size, PICOS, follow-up period) and provide the citations.	5, 6
Risk of bias within studies	19	Present data on risk of bias of each study and, if available, any outcome level assessment (see item 12).	7, 8
Results of individual studies	20	For all outcomes considered (benefits or harms), present, for each study: (a) simple summary data for each intervention group (b) effect estimates and confidence intervals, ideally with a forest plot.	6-8
Synthesis of results	21	Present results of each meta-analysis done, including confidence intervals and measures of consistency.	6-8
Risk of bias across studies	22	Present results of any assessment of risk of bias across studies (see Item 15).	7, 8
Additional analysis	23	Give results of additional analyses, if done (e.g., sensitivity or subgroup analyses, meta-regression [see Item 16]).	7
**DISCUSSION**			
Summary of evidence	24	Summarize the main findings including the strength of evidence for each main outcome; consider their relevance to key groups (e.g., healthcare providers, users, and policy makers).	8-10
Limitations	25	Discuss limitations at study and outcome level (e.g., risk of bias), and at review-level (e.g., incomplete retrieval of identified research, reporting bias).	10
Conclusions	26	Provide a general interpretation of the results in the context of other evidence, and implications for future research.	10
**FUNDING**			
Funding	27	Describe sources of funding for the systematic review and other support (e.g., supply of data); role of funders for the systematic review.	1

Table S2. Technical characteristics and evaluation of the reliability of the strain measurements in the included studies.

Authors	Publication Year	Machine	Software	Guidelines for Measurements	Strain	Reliability for LV-GLS	
						Interobserver	Intraobserver
**Garcia-Alvarez A. et al**	**2011**	**Vivid 7; General Electric Healthcare, Milwaukee, WI, USA**	**EchoPAC PC**	**American Society of Echocardiography**	**Two-Dimensional**	**Not mentioned**	Not mentioned
Nascimiento CA, et al	2013	Vivid 7; General Electric Healthcare, Milwaukee, WI, USA	EchoPAC PC	American Society of Echocardiography	Two-Dimensional	Not mentioned	Not mentioned
Barbosa M. et al	2014	Vivid 7; General Electric Healthcare, Milwaukee, WI, USA	Echopac PC, Version 7.0.X; GE Healthcare, Fairfield, CT, USA	American Society of Echocardiography	Two-Dimensional	0.97	0.97
Gomes VA et. al	2016	Vivid 7; General Electric Healthcare, Milwaukee, WI, USA	EchoPAC PC	American Society of Echocardiography	Two-Dimensional	0.71	0.69
Lima MS. Et al	2016	Vivid 7; General Electric Healthcare, Milwaukee, WI, USA	EchoPAC PC, v. BT10	American Society of Echocardiography	Two-Dimensional	Not mentioned	Not mentioned
Lima MS. Et al	2016	Vivid 7; General Electric Healthcare, Milwaukee, WI, USA	EchoPAC PC, v. BT10	American Society of Echocardiography	Two-Dimensional	Not mentioned	Not mentioned

Santos Junior OR. Et al	2019	Vivid 7; General Electric Healthcare, Milwaukee, WI, USA	EchoPAC PC	American Society of Echocardiography	Two-Dimensional	Not mentioned	Single individual observer
Cianciulli TF. et al	2020	Vivid 7; General Electric Healthcare, Milwaukee, WI, USA	EchoPAC PC	American Society of Echocardiography and the European Association of Echocardiography	Two-Dimensional	0.94	0.95
Romano MMD. Et al	2020	Vivid E9 or S6 (GE Healthcare, Horten, Norway)	EchoPAC PC	Not mentioned	Two-Dimensional	0.99	0.99
Echeverria LE. et al	2020	Vivid S6 (GE Healthcare, Horten, Norway)	EchoPAC PC	American Society of Echocardiography and the European Association of Echocardiography	Two-Dimensional	Not mentioned	Single individual observer

Table S3. Meta-regression analysis evaluating variables associated with the global longitudinal strain in patients with the indeterminate form of Chagas Disease.

Variables	Number of studies	Regression coefficient	95% CI	p-value	I^2 for heterogeneity (%)	R-squared (%)
Age	9	−0.31	−0.22, 0.16	0.743	97.18	0.01
Proportion of males	9	0.09	0.04, 0.15	**0.001**	92.11	58.37
LVEF	4	−0.05	−0.79, 0.68	0.885	93.37	0.00
LAVI	-	-	-	-	-	-
LVS	4	−0.11	−0.58, 0.35	0.627	91.83	0.01
LVD	4	0.05	−0.38, 0.49	0.793	94.25	0.01
E/e′ ratio	-	-	-	-	-	-

LVEF: left ventricular ejection fraction; LAVI: left atrial volume index; LVS: left ventricular stiffness; LVD: left ventricular dysfunction; E/e′ ratio: ratio of transmitral blood velocity to tissue Doppler velocity.

Table S4. Pooled mean values of segmental longitudinal strain in patients with Chagas Disease and healthy controls.

Variables	CCM Patients	NS	IF Patients	NS	Healthy controls	NS
Basal-Anterior	−17.8 (−24.7, −10.9. I^2: 17%)	4	−18.3 (−21.6, −15.1. I2: 0%)	6	−19.9 (−22.8, −17.1. I2: 45.5%)	5
Basal-Anterolateral	−19.8 (−29.6, −10.1. I^2: 7%)	2	−20.3 (−25.4, −15.2. I2: 0%)	3	−19.2 (−23.6, −14.9. I2: 0%)	3
Basal-Inferolateral	−14.8 (−24.9, −4.7. I2: 0%)	2	−18.9 (−24.1, −13.7. I2: 0%)	4	−19.6 (−23.7, −15.5. I2: 0%)	3
Basal-Inferior	−17.1 (−22.5, −11.5. I2: 0%)	4	−19.3 (−22.3, −16.3. I2: 0%)	6	−19.9 (−21.3, −18.6. I2: 0%)	5
Basal-Inferoseptal	−14.9 (−24.6, −5.4. I2: 50%)	2	−15.9 (−19.3, −12.6. I2: 0%)	4	−17.9 (−18.9, −17.1. I2: 0%)	4
Basal-Anteroseptal	−16.2 (−27.3, −10.2. I2: 0%)	3	−17.3 (−20.7, −13.8. I2: 0%)	5	−18.6 (−20.4, −16.9.I2: 0%)	4
Mid-Anterior	−16.2 (−21.7, −10.7. I2: 0%)	4	−18.5 (−21.7, −15.3. I2: 0%)	6	−19.6 (−20.9, −18.4. I2: 0%)	5
Mid-Anterolateral	−16.5 (−24.8, −8.2. I2: 0%)	2	−18.6 (−23.1, −14.1. I2: 0%)	3	−18.9 (−22.7, −15.1. I2. 0%)	3
Mid-Inferolateral	−15.2 (−22.7, −7.7. I2: 0%)	3	−18.6 (−22.3, −14.9. I2: 0%)	4	−19.4 (−23.0, −15.8. I2: 0%)	3
Mid-Inferior	−18.3 (−23.4, −13.1. I2: 0%)	4	−20.2 (−23.2, −17.2. I2: 0%)	6	−19.9 (−21.7, −18.0. I2: 20.6%)	5
Mid-Inferoseptal	−19.8 (−24.9, −14.8. I2: 0%)	2	−18.9 (−21.9, −15.8. I2: 0%)	4	−19.3 (−20.8, −17.7. I2: 0%)	4
Mid-Anteroseptal	−18.5 (−24.8, −12.2. I2: 0%)	3	−19.9 (−23.4, −16.5. I2: 0%)	5	−21.4 (−23.5, −19.4. I2: 0%)	4
Apical-Anterior	−14.9 (−22.4, −7.6. I2: 0%)	4	−19.3 (−24.5, −14.1. I2: 0%)	6	−19.9 (−23.0, −16.8. I2: 21.6%)	5
Apical-Lateral	−14.9 (−19.1, −10.7. I2: 0%)	3	−17.4 (−21.2, −13.8. I2: 29%)	4	−18.5 (−23.6, −13.5, I2: 81.9%)	3
Apical-Septal	−15.9 (−19.3, −12.6. I2: 0%)	3	−20.9 (−24.4, −17.3. I2: 0%)	3	−18.8 (−21.7, −16.0. I2: 0%)	2
Apical-Inferior	−20.6 (−26.8, −14.4. I2: 0%)	4	−23.6 (−27.5, −19.7. I2: 0%)	6	−20.9 (−23.6, −18.2. I2: 40.9%)	5

CCM: Chronic Chagas Cardiomyopathy; IF: Indeterminate form of Chagas Disease; NS: Number of studies.

Table S5. Meta-regression analysis evaluating variables associated with the global longitudinal strain in patients with chronic Chagas cardiomyopathy.

Variables	Number of studies	Regression coefficient	95% CI	p-value	I^2 for heterogeneity (%)	R-squared (%)
Age	8	0.22	-0.20, 0.64	0.305	97.84	1.02
Proportion of males	8	0.22	0.08, 0.36	**<0.001**	94.81	60.64
LVEF	8	-0.32	-0.47, -0.17	**<0.001**	92.71	70.88
LAVI	4	1.59	0.83, 2.37	**<0.001**	91.77	85.33
LVS	8	0.37	0.10, 0.63	**0.007**	95.64	49.63
LVD	8	0.15	-0.16, 0.47	0.342	97.95	0.01
E/e′ ratio	6	0.15	-1.01, 1.31	0.797	98.97	0.00

LVEF: left ventricular ejection fraction; LAVI: left atrial volume index; LVS: left ventricular stiffness; LVD: left ventricular dysfunction; E/e′ ratio: ratio of transmitral blood velocity to tissue Doppler velocity.

Table S6. Standardized mean differences in segmental longitudinal strain between patients with Chronic Chagas Cardiomyopathy, Indeterminate Form patients, and Healthy Controls.

Variables	CCM vs IF	NS	IF vs Healthy controls	NS
Basal-Anterior	0.46 (-0.19, 1.11. I^2: 90%)	4	0.22 (-0.01, 0.44. I^2: 28%)	5
Basal-Anterolateral	0.19 (-0.42, 0.80. I^2: 67%)	2	-0.06 (-0.30, 0.19. I^2: 0%)	3
Basal-Inferolateral	0.81 (0.28, 1.35. I^2: 82%)	3	0.02 (-0.54, 0.58. I^2: 79%)	3
Basal-Inferior	0.63 (0.19, 1.07. I^2: 77%)	4	0.25 (-0.05, 0.54. I^2: 55%)	5
Basal-Inferoseptal	0.36 (-0.58, 1.31. I^2: 86%)	2	0.49 (0.24, 0.74. I^2: 24%)	4
Basal-Anteroseptal	0.23 (-0.32, 0.79. I^2: 84%)	3	0.26 (-0.08, 0.59. I^2: 59%)	4
Mid-Anterior	0.61 (0.15, 1.08. I^2: 80%)	4	0.49 (-0.03, 1.02. I^2: 86%)	5
Mid-Anterolateral	0.46 (-0.10, 1.02. I^2: 60%)	2	0.09 (-0.16, 0.33. I^2: 0%)	3
Mid-Inferolateral	0.68 (-0.19, 1.55. I^2: 93%)	3	0.39 (-0.19, 0.97. I^2: 81%)	3
Mid-Inferior	0.67 (0.06, 1.27. I^2: 88%)	4	0.04 (-0.35, 0.42. I^2: 74%)	5
Mid-Inferoseptal	0.09 (-0.95, 1.15. I^2: 89%)	2	0.28 (0.05, 0.50. I^2: 10%)	4
Mid-Anteroseptal	0.50 (-0.05, 1.05. I^2: 83%)	3	0.32 (-0.22, 0.85. I^2: 83%)	4
Apical-Anterior	0.69 (0.09, 1.29. I^2: 88%)	4	0.31 (-0.15, 0.76. I^2: 81%)	5
Apical-Lateral	0.97 (0.35, 1.59. I^2: 80%)	3	0.79 (-1.71, 3.28. I^2: 98%)	3

Apical-Septal	1.45 (0.82, 2.09. I^2: 78%)	3	-0.53 (-1.26, 0.19. I^2: 73%)	2
Apical-Inferior	0.74 (0.05, 1.43. I^2: 90%)	4	-0.05 (-0.45, 0.36. I^2: 77%)	5

CCM: Chronic Chagas Cardiomyopathy; IF: Indeterminate form of Chagas Disease; NS: Number of studies.

Table S7. Sensitivity analysis assessing longitudinal strain differences between patients with Chronic Chagas Cardiomyopathy, Indeterminate Form patients, and healthy controls using mean differences.

Variables	CCM vs IF	NS	IF vs Healthy controls	NS
GLS	4.12 (1.67, 6.58. I^2: 96%)	7	0.45 (-0.45, 1.36. I^2: 85%)	8
Basal-Anterior	3.51 (-0.85, 7.87. I^2: 92%)	4	0.82 (0.23, 1.40. I^2: 0%)	5
Basal-Anterolateral	1.48 (-3.03, 5.99. I^2: 71%)	2	-0.21 (-1.24, 0.82. I^2: 0%)	3
Basal-Inferolateral	7.34 (1.24, 13.44. I^2: 92%)	3	-0.05 (-2.83, 2.73. I^2: 81%)	3
Basal-Inferior	3.63 (0.32, 6.94. I^2: 91%)	4	0.77 (-0.13, 1.67. I^2: 57%)	5
Basal-Inferoseptal	1.73 (-2.57, 6.02. I^2: 88%)	2	1.59 (0.78, 2.39. I^2: 29%)	4
Basal-Anteroseptal	1.66 (-1.89, 5.21. I^2: 91%)	3	0.86 (-0.15, 1.88. I^2: 49%)	4
Mid-Anterior	3.45 (0.33, 6.58. I^2: 89%)	4	1.72 (-0.06, 3.49. I^2: 86%)	5
Mid-Anterolateral	2.65 (-1.21, 6.51. I^2: 74%)	2	0.35 (-0.57, 1.27. I^2: 0%)	3
Mid-Inferolateral	5.22 (-1.73, 12.17. I^2: 97%)	3	1.26 (-0.65, 3.18. I^2: 77%)	3
Mid-Inferior	4.05 (-0.02, 8.11. I^2: 94%)	4	0.11 (-1.10, 1.32. I^2: 75%)	5
Mid-Inferoseptal	0.84 (-3.36, 5.03. I^2: 87%)	2	0.78 (0.13, 1.43. I^2: 18%)	4
Mid-Anteroseptal	3.08 (-0.86, 7.03. I^2: 92%)	3	1.05 (-0.67, 2.76. I^2: 82%)	4
Apical-Anterior	5.58 (0.30, 10.86. I^2: 92%)	4	1.69 (-0.89, 4.28. I^2: 83%)	5
Apical-Lateral	6.58 (-0.55, 13.71. I^2: 97%)	3	1.60 (-3.16, 6.36. I^2: 98%)	3
Apical-Septal	9.13 (2.88, 15.38. I^2: 94%)	3	-1.46 (-2.22, -0.71. I^2: 0%)	2
Apical-Inferior	6.30 (-0.28, 12.88. I^2: 96%)	4	-0.06 (-1.81, 1.68. I^2: 77%)	5

CCM: Chronic Chagas Cardiomyopathy; IF: Indeterminate form of Chagas Disease; NS: Number of studies.

Table S8. Methodological quality of included studies.

Authors	Publication Year	Design	Selection				Comparability	Outcome			Total
			1	2	3	4	1	1	2	3	

**Garcia-Alvarez A. et al**	**2011**	**Cross-sectional**				XX		XX	X	NA	5
Nacimiento CA, et al	2013	Cohort		x	X	X	X	X			5
Barbosa M. et al	2014	Cross-sectional				XX		XX	X	NA	5
Gomes VA et. al	2016	Cross-sec-tional*				XX	X	XX	X	NA	6
Lima MS. Et al	2016	Cross-sectional				XX	X	XX	X	NA	6
Lima MS. Et al	2016	Cross-sectional				XX	XX	XX	X	NA	7
Santos Junior OR. Et al	2019	Cohort			X	X	XX	X		X	6
Cianciulli TF. et al	2020	Case-Control	X			X	X	X	X	X	6
Romano MMD. Et al	2020	Cross-sectional		X		XX	X	XX	X	NA	7
Echeverria LE. et al	2020	Cross-sectional				XX	XX	XX	X	NA	7

Cross-Seccional (max 10 stars); Cohort/Case-control (max 9 stars); *Nested in a cohort.

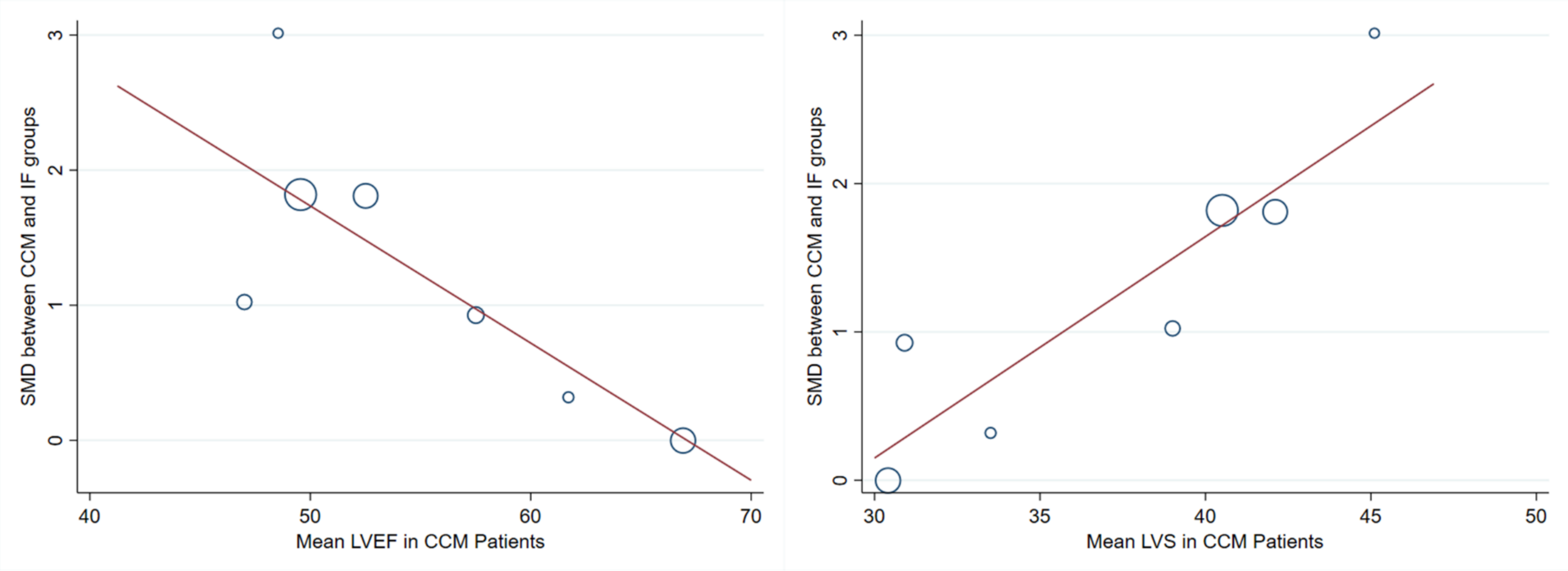

Figure S1. Meta-regression analyses evaluating the echocardiographic variables associated with the mean difference of the GLS observed between CCM and IF patients.

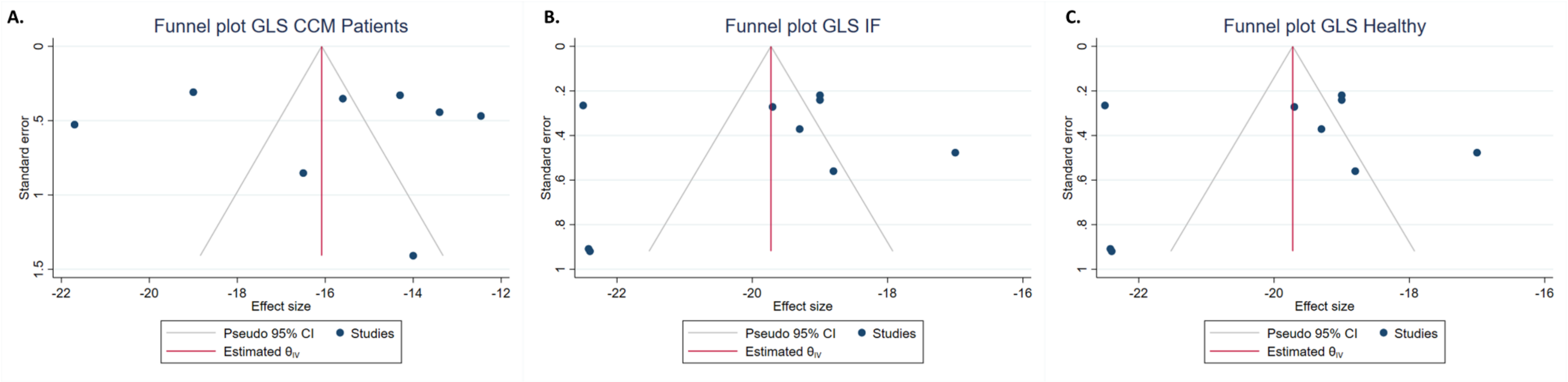

Figure S2. Funnel plots assessing the potential of publication bias for A.) Global longitudinal strain (GLS) in Chronic Chagas Cardiomyopathy, B.) GLS in patients with the indeterminate form of Chagas Disease, and C.) GLS in healthy controls.

Box S1. Search strategy.

Medline<br><br>TX (chagas disease OR T. cruzi OR Chagas Cardiomyopathy) AND TX (echocardiography or echocardiogram or speckle tracking strain)
EMBASE<br><br>('chagas disease':ab,ti OR 'trypanosomatid infection':ab,ti OR 'chagas cardiomyopathy':ab,ti OR 't. cruzi':ab,ti) AND (echocardiography:ab,ti OR 'transthoracic echocardiography':ab,ti OR echocardiographic:ab,ti OR 'systolic dysfunction':ab,ti OR 'speckle tracking echocardiography':ab,ti OR 'speckle tracking imaging':ab,ti OR 'heart function':ab,ti OR 'cardiovascular imaging':ab,ti OR 'ejection fraction':ab,ti)
LILACS

(Chagas disease [Palavras] or Chagas cardiomyopathy [Palavras]) and echocardiography [Palavras]
